# Characterizing the role of bots’ in polarized stance on social media

**DOI:** 10.1007/s13278-022-00858-z

**Published:** 2022-02-04

**Authors:** Abeer Aldayel, Walid Magdy

**Affiliations:** School of Informatics, University of Edinburgh, Edinburgh, UK

**Keywords:** Stance, Bots, Social media

## Abstract

There is a rising concern with social bots that imitate humans and manipulate opinions on social media. Current studies on assessing the overall effect of bots on social media users mainly focus on evaluating the diffusion of discussions on social networks by bots. Yet, these studies do not confirm the relationship between bots and users’ stances. This study fills in the gap by analyzing if these bots are part of the signals that formulated social media users’ stances towards controversial topics. We analyze users’ online interactions that are predictive to their stances and identify the bots within these interactions. We applied our analysis on a dataset of more than 4000 Twitter users who expressed a stance on seven different topics. We analyzed those users’ direct interactions and indirect exposures with more than 19 million accounts. We identify the bot accounts for supporting/against stances, and compare them to other types of accounts, such as the accounts of influential and famous users. Our analysis showed that bot interactions with users who had specific stances were minimal when compared to the influential accounts. Nevertheless, we found that the presence of bots was still connected to users’ stances, especially in an indirect manner, as users are exposed to the content of the bots they follow, rather than by directly interacting with them by retweeting, mentioning, or replying.

## Introduction

Social media platforms are used as a primary source for the dissemination of news (Center 2013). A vast number of users who depend on these platforms as their primary source of information also use them as a medium to express their stances on various events. A stance is defined as a person’s attitude and view toward an entity or subject, either in support or opposition (AlDayel and Magdy 2019a).

Social media platforms are infested with social bots^1^ (automated accounts) that mimic human behavior and can be used to spread inflammatory content with the aim of promoting a specific view or stance (Shao et al. 2018; Bessi and Ferrara 2016).

Due to the prevalence of bots on social media, humans are not the only players on these platforms, and bots have commonly been used to manipulate views by posting content and interacting with real users (Mendoza et al. 2020; Bessi and Ferrara 2016; Boichak et al. 2018). For example, these programs were used during the 2016 US presidential campaign to manipulate discussions by spreading content related to the US elections (Rizoiu et al. 2018; Ratkiewicz et al. 2011). In addition, in a recent study by Dunn et al. (2020), it shows that bots were used to spread fake news about Coronavirus (COVID19) in social media. All of these factors highlight the need to identify the role bots play in affecting the stance of social media users.

There is no concrete method to analyze the role of bots in affecting the social media users’ stances (Garimella and West 2019; Pulido et al. 2018). Nevertheless, there have been several attempts to gauge the effect of bots on various events such as elections (Boichak et al. 2018; Santia et al. 2019; Shao et al. 2018). The focus of these studies was to evaluate content diffusion on social networks as a way to measure the influence of bots on public stance towards a topic. Most of these studies evaluated the spread of the misleading and false information by social bots to measure the effect of these accounts on the discussion of an event (Tardelli et al.; Santia et al. 2019; Shao et al. 2018); for instance, study by  (Santia et al. 2019) and  (Tardelli et al.) evaluated the spread of misleading content on Facebook by bots, and spreading misinformation related to financial topics, respectively. Similarly, the work of Shao et al. (2018) found that bots amplified the spread of fake news within a 10-month period between 2016 and 2017. Previous studies used the spread of bots on social networks as indicators of their effect on social media user’s stances. While this method showed that bots are heavily present in social networks, there are still limitations when it comes to identifying whether the presence of bots is correlated with users’ stances towards specific topics.

In this paper, we seek to understand the interplay between bots and support/against stances with respect to a given topic. We study bots’ role and define their connection with stance interactions as the signals in the online social network that can be predictive for stance towards a given topic (AlDayel and Magdy 2019b). Our main hypothesis is that if bots exist among the most influential features for predicting the user’s stance, then it can be inferred that these bots have a role in pushing and/or reinforcing that stance.

Our approach for measuring the relationship between bots and online stance interactions is to build stance-detection models that are trained on users’ online social networks, then analyze the presence of bots among the most important features for predicting stance. Stance detection is a well-known task that has been used in multiple studies to infer a user’s leaning/ views towards an entity or topic. Many studies have used stance prediction to analyze the public view towards an event or entity by analysing social media users interactions (Magdy et al. 2016a; Darwish et al. 2017; Graells-Garrido et al. 2020).

### Hypotheses and research questions

Previous studies on the role and effect of bots on online social networks (OSN) highlight the need to address human interactions to effectively differentiate between automated and real accounts (Abokhodair et al. 2015).

In this paper, we investigate the following research questions:

1. Do social bots have a presence among the most influential network signals that can predict a user’s stance?
2. Is the interaction of social bots with users’ stances similar on both those with supporting stances and those with opposing stances? Or do they usually have a more noticeable relationship in a particular direction? Does this change according to the topic?
3. How does the relationship between the presence of bots and users’ stance change based on the type of interactions between the bots and the users? Do users directly interact with bots by retweeting/replying, or only by being exposed to their content by following bot accounts?

To this end, we proposed three hypotheses to assess bots’ interplay with specific stances, compared to real accounts. These hypotheses were derived from the induction phenomenon (also known as social influence)  (Friedkin and Johnsen 1990), wherein an individual’s behavior is affected by their social interactions (Gilani et al. 2019).

1. The presence of bots is an influential feature for predicting stance in an online social network.
2. For some topics, bots might have a presence in a certain stance over the other.
3. The specific type of bot interaction moderates the relationship between bot presence and stance.

To answer our previous research questions and test the previous hypotheses, we performed a large-scale analysis of bots on Twitter. We focused on Twitter,^2^ as this platform has been shown to be one of the popular social media environments for sharing content and spreading news. Also, many studies have indicated a substantial presence of bots on Twitter (Abu-El-Rub and Mueen 2019; Stella et al. 2018), which makes this platform suitable for our study. Then, we built a stance-detection model by using users’ interactions as the main features to infer those whose stances were in favor of and against a given topic. We used two types of online interactions, direct interaction (IN) and Indirect Exposure (EXP). The (EXP) interactions contain accounts collected from the user’s friends list (connection network (CN), while the direct interactions (IN) include a set of retweets, mentions, and replies in users’ tweets. By answering these research questions, we offer the following contributions:

- We assessed the interaction behavior of social bots to analyze their relationship with the *against* and *in-favor of* stance concerning specific topics.
- Most of the previous techniques for examining the effect of social bots evaluated synthetic datasets, and focused on the diffusion of these accounts in social networks. In this work, we provide a more robust technique to analyze the connection between bots and polarized stance interactions^3^ by using a gold standard stance dataset that covers various domains.
- We provide a comprehensive analysis of how users with specific stances were exposed to bot content through two types of interactions: (1) direct interactions (IN) through retweets, mentions, and replies to bots tweets, and (2) indirect interaction (EXP) by following bots and being exposed to their content.

We applied our experiments on a dataset of more than 4000 Twitter users who had expressed polarized stances towards seven different topics in multiple domains, including the political, social, and religious spheres. We analyzed those users’ online interactions and friendships with more than 19 million accounts, among which we identified the bot accounts and the ways in which the users of specific stance (favor/against) interacted with these bots accounts.

Our findings showed that a relationship between social bot accounts and users’ stances does exist, but it is minimal when compared to connections with human accounts, which were more significantly tied to user stance. We also found that the relationship between bots and user stance occurs when users follow the bot accounts and are exposed to their content; this effect was more apparent than the online signals coming from those users who directly interacted with those accounts through retweeting or replying.

## Related work

This section provides a discussion of previous work in inspecting bot’s effect on social media. Initially, we give some background on Twitter as a social media platform and its policy towards automated accounts, such as bots. Then, we show recent work on measuring the role of bots on the spread of discussions on social media. Finally, we discuss work related to inferring online signals on social media that are predictive of the stance towards a topic, which is our methodology’s primary instrument.

### Twitter policy on bots

Twitter is one of the largest online social networks (OSNs). Users can easily create an account, which is public by default, then they can follow any other public accounts without their consent. Only protected accounts, which are accounts that have their posts (tweets) seen only by their followers, are the ones that need explicit approval to follow them.

Unlike many of the social media platforms, Twitter allows accounts to post tweets automatically. This motivated many users and/or institutions to create bots, which are accounts that generate its content automatically and interacts on Twitter based on predefined rules  (Seering et al. 2018). Many bots accounts are created for useful causes, such as the Wikipedia edits bots “@*EarthquakeBot*”, which provides updates about earthquakes that measure 5.0 or more on the Richter Scale, as they happen.^4^ Twitter has a clear policy about the automation of accounts to regulate bots’ adoption on its platform.^5^ One of these rules is to prevent automated accounts from spamming the users or sending unsolicited messages.

Unfortunately, not all automated Twitter accounts (bots) got created for a noble cause. As will be discussed in the next section, some bots get created to spread fake news (Shao et al. 2018) or to create campaigns against election candidates (Bessi and Ferrara 2016), or to amplify specific stance on a topic (Stella et al. 2018). Thus, it became a crucial task for many researchers to build methods to identify bots and measure their spread in social networks. While there are quite many studies in these directions, there is still a limited amount of work to gauge if their role on stance is of any effect. In this study, we fill in the gap by investigating the bots interplay with stance.

### Bots’ role in online social networks

Bots have been always created and used on social media platforms for various good reasons, such as providing services/information on given subjects (Wate 2021) or for example, to help institutions with answering customer queries by text generating appropriate content  (Seering et al. 2018). Nonetheless, recent research brought awareness of the issue of bots on social media, where these automated accounts might usually employed for disruptive and malicious reasons such as spreading misinformation and spam messages  (Stella et al. 2018). This kind of bots have been associated with the risks of manipulating public opinion by artificially magnifying specific message on social media by retweeting specific message or spreading hateful posts  (Luceri et al. 2019). Most of the previous studies assessed the effect of bots by analyzing the spread of these accounts on social media as related to specific events  (Aiello et al. 2012; Abokhodair et al. 2015; Bastos and Mercea 2019; Ferrara 2017). For example, a study conducted by Rizoiu et al. (2018) used retweet diffusion to analyze the presence of bots in the first US presidential debate in 2016. They used synthetic data and generated an artificial social group of 1000 users to model cascades of retweets diffusion and to calculate users’ importance. Also, the study by (Ratkiewicz et al.) examined the diffusion of memes by bot account. In their study they analyzed political memes in Twitter and focus on detect astroturfing campaigns in the context of U.S. political elections. The work of (Hegelich and Janetzko 2016) investigated bot activity in the Ukrainian-Russian conflict and concluded that autonomous bot behavior helped spread content. A study analyzed the spread of bots in discussions related to the Syrian civil war by using 3000 tweets related to the topic  (Abokhodair et al. 2015). They found that the growth and content of botnets did not aligned with the bots main behaviour as these bots were spamming the hashtags with topics not related to war. Another study by Bastos and Mercea (2019) analyzed the bots behavior in Brexit discourse on Twitter  (Bastos and Mercea 2019). In their study, they used retweets to inspect user-to-bot and bot-to-bot cascade composition. They found that a botnet spread content supporting the “Leave” campaign. The study of  (Stella et al. 2018) evaluated the role of bots in spreading negative content according to social media data. In their study, they collected data related to the 2017 Catalan referendum and analyzed the diffusion of negative content by bots. They used Logistic Regression (LR) along with accounts metadata to identify bots accounts. Their results showed that bots increased the exposure to negative content. Along the same line, the study by  (Luceri et al. 2019) estimated the stance of bots on social media according to the content they spread. A recent work by  (Mendoza et al. 2020) focused on retweet only and analyze the presence of bots. Their findings show that there are different degrees of integration with different types of users. Another study by  (Ng and Carley 2021) studied the changing stance on a specific topic (coronavirus vaccine) using Twitter data from April 2020 to May 2021. In their study, they found that a larger proportion of bots are more prone to flipping their stances compared to non-bot accounts, where these normal accounts did not change their stance.

Another study by Abu-El-Rub and Mueen (2019) analyzed bot behavior in social media related to the US election and quantified the level of bots and human participation in social campaigns. By analyzing the retweets network, they found that bots’ interactions can corrupt social campaigns. Also,  (Schuchard et al. 2019) examined bots’ activities on twitter concerning the US 2016 elections and concluded that bots tend to have a hyper-social nature. Along the same lines,  (Gilani et al. 2019) provided a comparison between bots and human behavior with a focus on online activity. They used manual annotations to label the accounts as a bot or not. In their study, they showed that humans have a higher follower rate compared to bots. On the same line, a study by ‘(Aiello et al. 2012) used bots accounts to suggest friendship to Twitter users. They finding indicates the importance of trustworthiness as in general people are aware of the presence of the bot have been more inclined to follow its suggestions.

Another line of studies analyzed bots behavior on different kinds of platforms, such as Twitch and Wikipedia. For example,  (Seering et al. 2018) analyzed the social actions of bot services on the Twitch platform; in this study, they limited their analysis to the service bots provided for Twitch users. Another study analyzed the role of bots on Wikipedia and studied the editing behavior thereof and the effect on human editors; they found that the overall human interactions with bots were more in comparison with bots, compared to human-human interactions.

Most of the previous studies examined the effects of social bots by measuring their presence and the spread of their content on social networks. However, there is a gap in the literature to understand if the spread of these bots has a presence within the signals that predict users’ stances. Our study extended the efforts to assess the relationship between bots and users’ stance on social media by assessing the interplay between bots and users’ stances. Moreover, in contrast to previous studies, we provide a fine granularity analysis of bots on polarized stances (i.e., against or in favor). We utilized the advances in stance-detection models using network features to measure bots’ presence in the signals that are predictive for stance. Our novel approach states: the more bots that are present among the top predictive features for a specific stance, the stronger the relation between the presence of bots and a given stance of users on the topic.

### Online factors influencing the stance prediction on Twitter

A large body of work has analyzed the stance towards a topic on Twitter. Most of the work in this realm studied public stances in the aftermath of an event and analyzed the interplay between these stances and other online factors; for example, the work of (Allcott and Gentzkow 2017) showed the interplay between polarized stances and the sharing behavior of unreliable news content. Another work by Graells-Garrido et al. (2020) showed the temporal effect of online interactions on discussions related to abortion. The work by  (Musco et al. 2018) examined the trade-off between disagreement and polarization using synthetic along with Riddet data. The study demonstrates that overall users prefer links that minimize disagreement due to the well-known confirmation bias. Moreover, the study by AlDayel and Magdy (2019b) shows that user’s interactions and connection networks had the best performance when it came to predicting the user’s stances, compared to textual features; they built a stance-detection model using three different kinds of the networks: an interaction network, a preference network, and a connection network. Their findings highlighted that a user’s online interactions or connections with other accounts were effective signals to predict their stances towards different topics. A recent survey (AlDayel and Magdy 2021) provides a thorough overview of the most predictive features for stance detection in social media. It lists multiple studies that confirm the effectiveness of network interactions as a predictor of users’ stance in social media.

## Data collection

To examine the role of bots on users’ stances, we utilized datasets that contain ground truth labels for stances on seven topics. This section provides a description of the datasets used and explains the process of constructing users’ networks.

### Stance-detection datasets

We used two datasets that contain tweets that are labeled for stances towards seven topics. These datasets are:

**SemEval stance dataset**. We chose this dataset because it is considered to be one of the most well-known stance dataset that covers topics from different domains. A recent study by (AlDayel and Magdy 2019b) used SemEval stance data to provide a thorough assessment of social media online signals that affect stance prediction. This dataset contains tweets related to five topics: Hillary Clinton (HC), Climate Change is a real concern (CC), the Feminist Movement (FM), Legalization of Abortion (LA), and Atheism (A) (Mohammad et al. 2016). The dataset is publicly available and consists of 4,163 tweets collected in 2016 that are labeled with three classes of stances: in-favor, against, and either. Since the focus of this study was to investigate the relationship between social bots and users’ stances, we used the “Users Dataset,” a subset of the SemEval dataset that was published by (AlDayel and Magdy 2019b). This dataset contains 2,875 tweets, which are a subset of tweets from user accounts that are still available on Twitter.

**Events dataset**. We created an additional dataset that covered two recent topics: Brexit (B) and Immigration (I). These topics were selected because they were one of the viral events at the time we were collecting data. The tweets in this dataset were all selected to be replies to other tweets to have a higher chance of showing a polarized stance as being part of a discussion. We collected tweets on Brexit (B) in February 2019 using the keyword “Brexit.” For the topic of Immigration, we collected the tweets in October 2018 using the following keywords: “immigrant,” “refugee,” and “border.” Tweets of both topics were submitted to the crowd-sourcing platform Appen.^6^ We followed the same annotation guidelines used to construct the SemEval stance dataset (Mohammad et al. 2016), and each tweet was annotated as “favor,” “against,” by five annotators while taking the majority vote as the final label for each tweet. For each topic we created around 100 quality control test instances (labels for a given tweet) to verify the annotators ability of assigning the correct labels.^7^ These test instances with pre-answered labels are used to further qualify high-performing contributors, remove under-performing ones, and continually training contributors to improve their understanding of the task. Furthermore, we choose the high qualified annotators in Appen and accept Level 3 annotators for the task to ensures only our most experienced and highest performers will work on your task. The inter-annotator agreement between the annotators for Brexit was 73%, and the score for Immigration was 75%; these scores demonstrate a high level of agreement between the annotators, which indicates that different annotators frequently gave the same response (stance) for the same tweet.

Table 1 shows sample of the tweets in our data collection along with their stance labels to the corresponding topic.

### Collecting users’ online interactions

For each tweet in our dataset, we collected all the interactions information for its author. For each user, we collected two types of online interactions, as defined by the work of  (AlDayel and Magdy 2019b). The first is $\hbox {IN}_@$, which is the interaction network of the user that includes all the accounts the user retweet, mention or reply; and the other is $\hbox {CN}_{{FR}}$, which is the connection network of the user that includes the list of accounts the user follows. We used Twitter API to collect users’ timelines, which included all the tweets they posted or retweeted in their home-timeline.^8^ From the timeline, we extracted all the accounts that the user retweeted, replied, or mentioned to represent the $\hbox {IN}_@$. We also collected the friends list (i.e., the accounts the user follows) using Twitter API .^9^

Table  11 shows all statistics related to our datasets, including the number of tweets and users for each of the seven topics, which are labeled according to stance, and the number of collected accounts in the interaction and connection networks. As shown in the table, the total number of accounts collected for all the users in our datasets was more than 19 million accounts, which means that on average, each user interacted and/or connected with more than 4000 accounts in total. The median number of accounts the user interacts with (IN) is 1,288 (average = 2,532), and the median number of accounts the user follows ($\hbox {CN}_{{FR}}$) is 602 (average = 2,101).

Our aim in this study is to identify which of those accounts are bots and to understand which of those are shown to have predictive features for specific stances; in this way, we can explore the relationship between bots and user stances online (Table 2).

**Table 1:** Sample tweets from each dataset

#	Tweet	Topic	Stance
1	Those of us that also have a brain will be voting against Hillary	HC	Against
2	The carbon clock is ticking @CarbonBubble #SurplusGas #carbon #gas	CC	Favor
3	Close the border with military or any means possible. If we don’t STOP IT NOW, it will over run our country to the point of no return. We have to send a strong message and not back down!	IM	Against
4	Yes it is. But how long do we want to play this game? Enough is enough. #Brexit means Brexit, nobody voted for a deal, and so on and so forth. The UK just needs to leave on 29th of March.	B	Favor

Topics: hillary clinton (HC), climate change is a real concern (CC), immigration (IM), and brexit (B)

**Table 2:** The number of tweets per topic in the SemEval and Events datasets with the number of unique users who authored the tweets shown in brackets

Dataset	Topic	Tweets (users)	IN$_{@}$	CN$_{FR}$
SemEval	Atheism (A)	550 (426)	608,399	740,878
	Climate change (CC)	461 (381)	560,629	524,591
	Hillary clinton (HC)	670 (511)	1,151,355	1,217,426
	Feminist movement (FM)	524(441)	657,411	371,700
	Legalization of abortion (LA)	670 (490)	978,300	938,184
Events	Brexit (B)	466 (466)	2,129,244	656,864
	Immigrations (I)	1512 (1512)	5,567,226	3,274,835
	Total	4853 (4227)	11,652,564	7,724,478

The total number of accounts users interacted with ($\hbox {IN}_@$) and followed ($\hbox {CN}_{{FR}}$) for each topic

## Assessing the role of social bots

This section describes the methodological framework that examined the connection between bots and users’ stances in social media. As mentioned earlier, our methodology for measuring the relationship between bots and users’ stances is by building a stance classifier using network features and inspecting bots’ presence within the most influential features. This section discusses our framework, which includes building an effective stance classifier, extracting the most predictive features for a given stance, and identifying the bots among the accounts.

### Stance detection classifier

Stance detection is a well-known task that has been used to infer a user’s leaning towards an entity or topic (Magdy et al. 2016a; Darwish et al. 2017; Samih and Darwish 2020).

For example, in their study, Grčar et al. (2017) built a stance classifier to analyze the stances toward the Brexit referendum using 37,000 tweets. Another study by Magdy et al. (2016a) analyzed the stances toward Muslims in the wake of the November 2015 Paris terrorist attacks. Moreover, (Magdy et al. 2016b) used a stance classifier to analyze the supporters of Hillary Clinton and Donald Trump in 2016 US elections.

A significant amount of work has studied stance detection by using network interactions to predict people’s leanings towards an event or entity (Thonet et al. 2017; Darwish et al. 2019). The study by Lai et al. (2018) used a social network community based on retweets, quotes, and replies to extract network-based features to train a stance-detection model. Similarly, a study by Darwish et al. (2019) designed an unsupervised stance-prediction model using a clustering algorithm with a combination of network features, namely retweeted tweets, retweeted accounts, and hashtags. These studies highlight the importance of the social network interactions of online users to detect their views in support of or opposed to specific topics.

The first step in our methodology was to build a stance classifier that classifies a given user’s stance as being in favor of or against a given topic.

To create an effective stance classifier, we replicated the current state-of-the-art stance-detection model devised by AlDayel and Magdy (2019b), which reported the best results-to our knowledge-on the SemEval stance dataset. We used a binary SVM with a linear kernel, and the parameters were tuned using fivefold cross-validation on the training set. In the study by AlDayel and Magdy (2019b), they showed that a binary classifier that is trained on the two classes of “in favor” and “against” while ignoring the “neither” class achieved a better performance than a three-class classifier. This setup was ideal for our purpose, since we were only focusing on the roles of bots on influencing stance and were thus not interested in the “neither” class ; consequently, we followed this same setup. A stance-detection model was trained for each topic separately, which means we trained seven different models for each of our seven topics.

Regarding the features, AlDayel and Magdy (2019b) compared multiple sets of features, including content features that are extracted from the text of tweets, different online interactions features of users’ accounts, and even the web domains that are linked in users’ tweets. To serve the purpose of our analysis, we focused on online network features; namely the interaction (IN) and connection (CN) network of users, both of which achieved the highest performance on the SemEVal dataset (AlDayel and Magdy 2019b).

We trained the stance detection model on two sets of features, and we refer to each of these as follows:

- *IN*, which included all the accounts with which each user directly interacts through retweets, replies, or mentions; also includes the website domains the users included in their tweets(AlDayel and Magdy 2019b).
- *EXP*, which corresponded to $\hbox {CN}_{{FR}}$ in (AlDayel and Magdy 2019b), and included the list of the accounts that the user followed. We called it *EXP*, since it represented the accounts the user was *exposed* to by following them, and were thus affected by their content, even without directly interacting with the content thereof by liking, replying, or retweeting.

For each topic, the labeled datasets were split into 70% for training and 30% for testing to build the stance classifier. This is the same split reported by the SemEval stance dataset  (Mohammad et al. 2016). For the Events dataset, we applied a random split to training and testing with the same split percentage-70% and 30%- for training and testing, respectively. The stance classifier was separately trained for each topic twice: once by using the IN features of each user, and another by using the EXP features thereof. For evaluating the classification performance, we used the SemEval stance-detection official evaluation script to calculate the F1-score (Mohammad et al. 2016) (Appendix A). The macro $\hbox {F}_{{avg}}$ is calculated as presented in Eq. 1.

1 $$\begin{aligned} F_{\mathrm{avg}} = \frac{F_{\mathrm{favor}} + F_{\mathrm{against}}}{2} \end{aligned}$$

The official evaluation measure used by Semeval2016 does not disregard the ‘none’ class. As by taking the average F-score for only the ‘favor’ and ‘against’ classes, we treat ‘none’ as a class that is not of interest or ‘negative’ class in Information Retrieval (IR) terms. Falsely labeling negative class instances still adversely affects the scores of this metric.

Table 3 shows the performance on the seven topics reported in the F1-score using the script provided by the SemEval task (Mohammad et al. 2016). As a validation for the model quality, our results for the five topics of SemEval aligns well with those reported in AlDayel and Magdy (2019b), which confirms that we succeeded in replicating the state-of-the-art stance detection model.^10^

**Table 3:** The average F1-score for stance detection on the seven topics in our two datasets

Topic	A	CC	HC	FM	LA	B	I
IN	71.9	48.2	71.8	61.2	70.3	47.6	55.8
EXP	68.05	48.21	72.98	66.0	66.42	69.2	49.00

### Extracting the most influential features on stance

To assess the extent of the relationship between bots and users’ stances, we analyze the most effective features for the stance prediction model. For each polarized stance (favor or against), we use the weight of the coefficient generated by the stance model to identify the set of the most influential features on the stance prediction. These features are extracted from the features set which contain the accounts and the domains (URLs) the user interacted within the (IN) feature set, and the accounts the user follows in the (EXP) feature set. We use the top 1000 most influential accounts for the stance prediction model from each feature set, excluding the domains from the IN features in our analysis, since our focus in this study is on bots’ connection to user stance.

In the next section, we inspect the population of bot accounts that exist in those 1000 most predictive accounts for user’s stance, and compare their population to other accounts.

### Identification the presence of bot accounts

In the next section, we inspect the population of bot accounts that exist in the 1000 most predictive accounts for a user’s stance, and compare this population to other accounts.

There is a large body of work focused on the development of techniques to detect bots in social media (Davis et al. 2016; Puertas et al. 2019; Santia et al. 2019). The work of (Puertas et al. 2019) used a multilingual classification model to identify bot accounts based on the content of their posts. Another work by Dutta et al. (2021) focused on the fraudulent user activities where they proposed a framework to detect collusive users involved in ’following’ activities. Similarly, the work by Cresci et al. (2015) introduced a framework to detect Fake followers on Twitter only. One of the most popular bot-detection APIs is Botometer^11^ (Davis et al. 2016; Yang et al. 2019), which provides a robust method to detect the existence of bots in social media. Botometer uses a Random Forest classification algorithm to classify tweets as bots based on 1000 features that were extracted from users’ meta data along with tweets timeline. The classification score ranges from 0 to 1, where 0 indicates the likelihood that the account is human and 1 indicates that the account is likely to be non-human (“*bot*”).

The Botometer API has been used in various studies to detect the existence of bots in the social media (Rizoiu et al. 2018; Varol et al. 2017; Broniatowski et al. 2018). In the study conducted by Rizoiu et al. (2018), the Botometer API was used to analyze the role and influence of bots on social media in the 2016 US Presidential Debate. Another study by Broniatowski et al. (2018) estimated the bot scores of Twitter accounts that spread content related to the vaccine debate on social media. Along these lines, we used Botometer in our study to detect the bots in our dataset.

To identify the bots in the set of predictive accounts extracted from the stance-detection model, we used the Botometer API (Davis et al. 2016). This API generates a score $\in [0,1]$, where 0 indicates the account of a real user, and 1 suggests the strong likelihood of a bot.

Sometimes the Botometer API generates an error message. This happened when it failed to access the tweets of an account because it is deleted, suspended, or protected. We considered protected accounts to be human accounts, since it was unlikely that a bot would restrict its tweets to only its followers (Rizoiu et al. 2018). While suspended accounts could be suspended because they were bots, Twitter can also suspend an account if the user of that account violates the platform rules.^12^ Common reasons to suspend a Twitter account includes abusive tweets, spamming, or if the account has been hacked or compromised. For the previous reasons, we did not consider the suspended accounts in our dataset to be bots; instead, we treated these accounts as “unknown” and labeled them as “deleted.” Therefore, the deleted and suspended accounts in our dataset were labelled as *“deleted”*.

For accounts that have a low botometer score, which are most likely to be non-bots (i.e., human accounts), we wanted to make a distinction between famous and normal accounts, since it might be expected that influential accounts will have a more prominent relationship with users’ stances than normal accounts. According to the research conducted by Cossu et al. (2016), the authors postulated that an account was more influential when it had more followers.

Thus, we further classified the non-bot accounts according to the number of followers thereof into three categories: ultra-famous, famous, and normal.

According to Twitter users statistics,^13^ only 0.05% of Twitter accounts have more than 10,000 followers; thus we label them as ultra-famous; the famous accounts are those with a number of followers ranging between 1000 and 10,000, and which applies to 2% of Twitter users; and finally, the normal accounts were those with fewer than 1000 followers, which applies to 98% of Twitter users.

## Results and analysis

In this section we assess the role of the social bots in detecting online stance by analyzing the top influential accounts that were the most predictive toward the polarized stances with regard to each topic.

### The distribution of bot scores of the most influential accounts

**Fig. 1:**
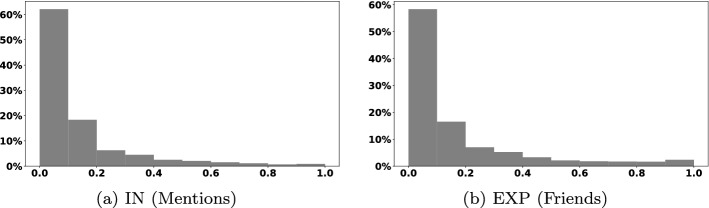
Botometer score distribution of the top 1000 accounts that are predictive to stance for both networks

Figure 1 shows the distribution of Botometer scores for the top 1000 accounts that are most predictive for stance in our dataset, for both the interaction and exposure network features (IN and EXP).^14^ The scores generated by Botometer are within a range $\in [0,1]$, where 0 indicates the account of a real user, and 1 suggests the strong likelihood of being bot.

As shown in Fig. 1, most of the accounts that are predictive to stance have a low bot score, where the majority of accounts have scores between [0, 0.2], indicating that these accounts are most like to be real people. Only very few accounts have high bot scores ( more than 0.6). This indicates that most of the accounts that have a role in predicting users’ stance are for real people.

To enable a more in-depth analysis for accounts that are more likely to be bots, we focused on those accounts that got a score of over 0.6, which indicates high likelyhood of being a bot, which is the same score used in previous studies to analyse bot behaviour (Rizoiu et al. 2018; Ferrara 2019). In these studies, an account was classified as a bot when the Botometer score exceeded a threshold of 0.6, where they showed that this score decreased misclassification and improved the overall bot-detection accuracy (Rizoiu et al. 2018). We followed the same setup and used the same threshold.^15^

The next section provides a further analysis of these accounts that are likely to be bots given their high bot score and compare them to human accounts (accounts with low Botometer score) and deleted accounts.

### The role of social bots on stances

For each type of feature (i.e., IN and EXP), we show the percentage of likely-bot accounts alongside other types of accounts for each topic.

**Fig. 2:**
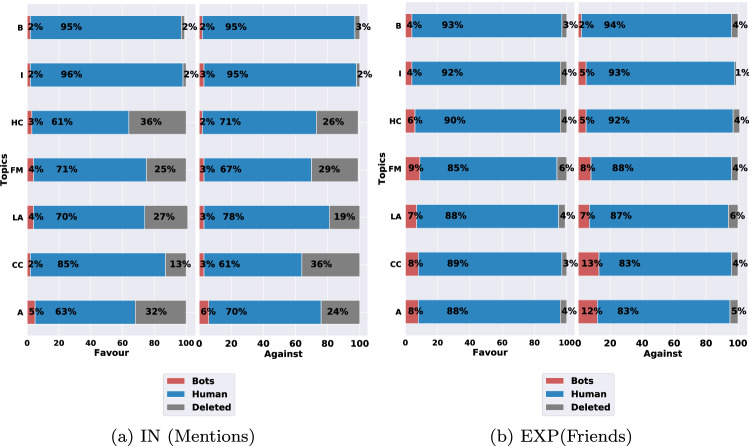
Distribution of social bots for each topic in the top 1000 most predictive accounts for polarized stances using direct interaction (IN) and indirect exposure (EXP) features. Atheism (A), Climate Change Is a Real Concern (CC), Hillary Clinton (HC), the Feminist Movement (FM), Legalization of Abortion (LA), Immigration (I), and Brexit (B)

**Direct interactions (IN).** Figure 2 shows the percentage of social bots on the in-favor or against stances with respect to the top 1000 IN features for each topic. The results show the prevalence of real accounts in the set of most influential features on the stances, compared to the minuscule existence of social-bot accounts. This trend is consistent in each topic with respect to both in-favor and against stances. The bots had an existence not exceeding 6% of the overall set of influential accounts in each topic, while non-bot accounts constituted the majority of the most influential accounts, reaching higher than 95% for some topics. As shown in Fig. 2a, some of the accounts in the top 1000 were deleted by the time we inspected them, especially those related to the SemEval stance dataset topics, since the data was more than four years old. This is one of the limitations of working with Twitter, since we cannot retrieve information from those accounts after deletion. This kind of limitation is well known in the online social network studies (Boichak et al. 2018; Ferrara 2017). Nevertheless, we still had the tweets wherein these accounts were mentioned in the collected users’ timelines, which allows us to provide further analysis to these accounts.

**Indirect exposure (EXP).** Figure 2b illustrates the percentage of bots in the favor or against stance with respect to the top 1000 EXP features for each topic. Again, the percentage of bots is minimal compared to human accounts. However, it is worth noting that bots constitute more population in the EXP network compared to IN, where it reaches 12% and 13% in some cases (Climate change and atheism topics). This suggests that being exposed to bots’ posts might be more strongly tied to users’ stance than direct interactions with bots.

Furthermore, these bots that people follow have a stronger connection to the *against* stance of some of the topics compared to the *favor* stance. For instance, users with against stance towards atheism tend to be affected by bots accounts more than users supporting Atheism (12% against vs. 8% in favor).

The same trend can be seen in climate change and immigration topics. This demonstrates that bots can have larger relationship to one stance direction compared to the other.

**Fig. 3:**
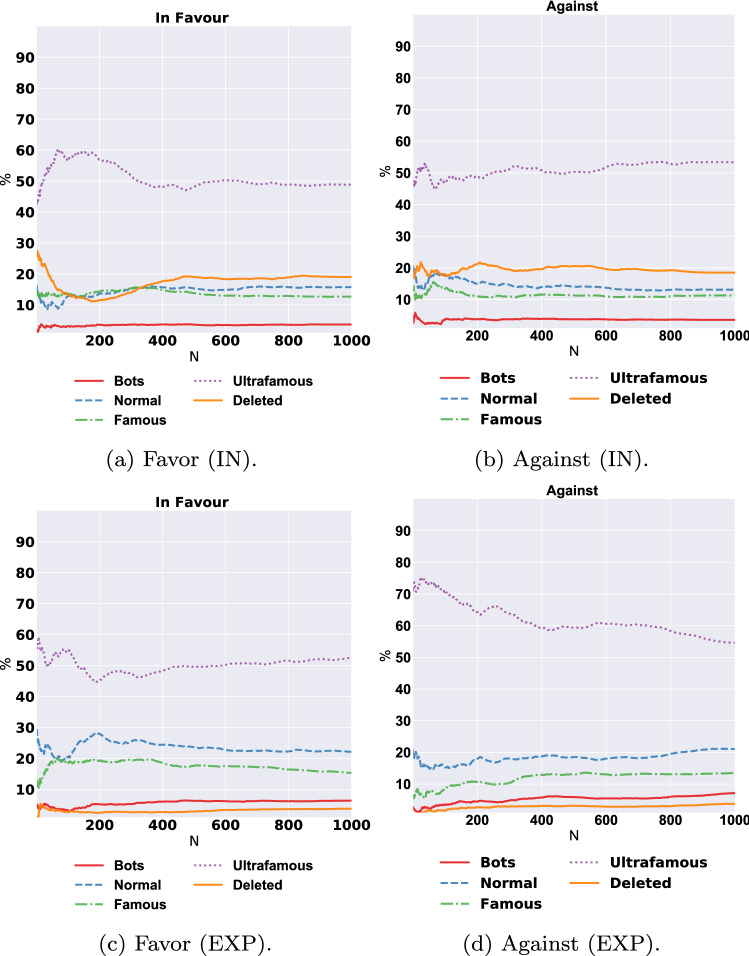
The percentage of each account type (*X*-axis) in the top N (*Y*-axis) important features of stance detection to predicting the Against/Favor stances in direct interactions (IN) and indirect interactions (EXP)

### Magnitude of the bots’ role

The previous analysis showed that the majority of the top 1000 predictive accounts of predicting stance for all the topics was for humans. However, a small proportion of bots might still be the most influential among those 1000. Thus, in this section, we provide a more in-depth analysis to the distribution of bots in the top *N* predictive accounts, where *N* ranged between 10 and 1000. In addition, we analyze the type of human accounts according to how famous they are.

Figure 3 illustrates the average distribution of different types of accounts on each polarized stance extracted from the IN and EXP networks averaged across all topics. We noticed that the distribution of bot accounts constituted the lowest percentage across all values of top *N* features. In fact, the average distribution of bots never exceeded 10% at any point. This was consistent across both networks and for both stances. The ultra-famous accounts, which were the human accounts with more than 10,000 followers, constituted the majority of accounts; they consistently constituted more than 50% across all values of *N* over all stances and networks, and their influence reached over 70% in the top 10 features for the EXP against stance. This means that following these accounts related to users’ stances being against a given topic.

Further detailed results for each of our seven topics are presented in Appendix C, where we show variations across some of the topics. For example, for Brexit, the ultra-famous users had a noticeable connection to the in-favor stance, reaching approximately 75% of the top 100 accounts, while the ultra-famous users only constituted 25% in the against stance. Moreover, for the against stance in EXP interactions, these accounts showed a sizable presence in the top 100 features for six topics: atheism, climate change, the feminist movement, Hillary Clinton, immigration, and Brexit. For the legalization of abortion, the normal accounts with fewer than 1000 followers had the most presence in the top 800 features of people who were opposed to the legalization of abortion.

Our analysis shows that bots have some role in relationship to online stance of being in-favor/against a topic, however to a much smaller degree than what we expected in our first hypothesis (H1). The relationship is minimal compared to that with human accounts, especially the ultra-famous accounts, which had the most association with users’ stances by far. We also found that bot accounts that people follow and are exposed to their content (EXP) has more influence ( presence in the top features), than bots with which users directly interacted. We applied a statistical significant test using Pearson’s chi-squared test between the distribution of bot accounts in the IN and EXP and found that bots presence in the EXP network is statically significantly higher than IN for all stances in most of the topics with *p*-value less than 0.001, except the Brexit and immigration topics, where both had the least number of bots (only 2–3%). Table 10 in Appendix D shows the full values of the chi-squared test per topic and stance. This result confirms our third hypothesis (H3) that people stances can be affected indirectly just by getting exposed to bots content, as we actually showed that EXP network affects users’ stance more than IN network.

**Fig. 4:**
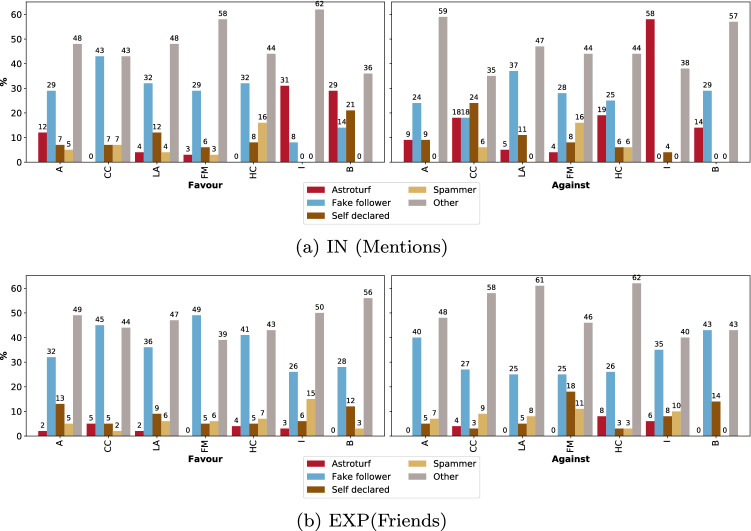
Distribution of social bots types for each topic in the top 1000 most predictive accounts for polarised stances using direct interaction (IN) and indirect interaction (EXP). Atheism (A), Climate change is a real concern (CC), Hillary Clinton (HC), Feminist movement (FM), Legalization of abortion (LA), Immigration (I), Brexit (B)

### Properties of the bot accounts

We further analyze the properties of the bots identified within the most predictive accounts of users’ stance, where we check their types and the number of followers they have in comparison to the human accounts.

**Types of the influential bots.** The new version of Botometer API (V4) provides the type of bot based on of six categories.^16^ These categories are: Astroturf, Fake follower, Financial, Self declared, Spammer and Other (miscellaneous). The *Astroturf* bots are political bots and accounts involved in follow trains that systematically delete content. The *Fake follower* bots are bots purchased to increase follower counts. *Financial bots* are the automated accounts that post using cashtags. The *Self declared* are bots labeled using botwiki.org, which is a website that keep track of useful and creative bot accounts that self-declare themselves as bots. While the *Spammer* are automated accounts labeled as spam bots from several datasets. Bots labeled as *’Other’* are the miscellaneous other bots obtained from manual annotation or reported by other users.

We used Botometer V4 to analyse the types of bots we identified in the influential accounts. Figure 4 shows the distribution of bots types (IN) and (EXP) networks. It can be noticed that the most dominating bots are the bots of type *Others*, that are obtained from manual annotation and user feedback. The Astroturf bots constitutes most of bots in the direct interactions networks that influence the against stance towards immigration and favor stance towards Brexit. Nonetheless, *Astroturf* bots shown to have the minimal presence in the indirect interactions (EXP). Bots that are identified as *Fake followers* have good presence in most topics, especially the exposure network. Overall, *spammer* bots constitute the minimal percentages over direct and indirect interactions, while *Financial* bots have no presence in the most influential accounts. These results show that bots that have an interplay role with stance are mostly the ones that got reported by normal users, while political bots (astroturf) still have some role, especially in stances on political topics.

**Followers of influential bots.** As our analysis has shown, ultra-famous accounts are the most influential in predicting stances. Thus, we further analyzed the bots’ number of followers to understand to compare them to the influential human accounts. Table 4 shows the split of the identified bot accounts by their number of followers as normal, famous, and ultra-famous. As shown, the majority of bots (50–60%) have less than 1000 followers. However, around 30–40% of them still have a large number of followers, especially for those bots influential to the against stance in the IN, around 34% of them are considered ultra-famous accounts by having over 10,000 followers. This shows that some of the bots that are popular on Twitter.

**Table 4:** Distribution of bots and human based on followers. The Ultra-famous accounts more than 10,000 followers; The famous accounts are those with number of followers ranging between 10,000 and 1000; The normal accounts less than 1000 followers

IN				
Account type	Favour		Against	
	Bots	Humans	Bots	Humans
Normal	57.73%	16.52%	51.57%	12.53%
Famous	19.58%	16.70%	14.73%	12.55%
Ultra-Famous	22.68%	66.77%	33.68%	74.90%

EXP				
Account type	Favour		Against	
	Bots	Humans	Bots	Humans
Normal	63.39%	21.71%	59.25%	20.63%
Famous	22.00%	16.66%	20.07%	14.68%
Ultra-Famous	14.59%	61.61%	20.66%	64.68%

### The context of the bots

In this section, we analyze the context of interactions between bots and users for each stance. We explore some examples of the bots appeared in users’ timelines to understand the possible link between the bots and users’ stances. We also check the nature of some of the bots that the users followed to estimate the exposure that might have affected their stance.

**The direct interaction with bots.** Table 5 presents a sample of the tweets generated by the bots that were the most predictive of the stance in the IN features. We found these bots in the users’ timelines as retweets, replies, or mentions. In general, bot interactions with social media users have three forms: 1) bots with content that aligned with the user’s stance; 2) bots with content that disagreed with the user’s stance; and 3) bots with content that had no relation to the user’s stance. For instance, the bot account “@*FollowDMs*” was one of the influential accounts in predicting the against stance to atheism, yet this account had no relation to the topics of atheism or religion (see Example 1). Additionally, regarding bot accounts that had no direct relation to immigration, the “@*cookequipmant*” account was one of the most influential accounts for predicting the stances related to the topic of immigration (see Example 7). Furthermore, Example 5 shows that users who supported atheism tended to directly interact with bot accounts that contradicted their stance. For example, one of the influential bot accounts in predicting the in-favor stance towards atheism was the one that promoted religious content, “@*BibleWisdoms*”. Additionally, Example 3 shows that the account “@*VoteHillary*2016” was a bot account that supported Hillary Clinton, yet it was one of the accounts that had a strong effect on predicting the against stance to Hillary Clinton. Moreover, users with an against stance to atheism interacted with religious bot accounts that promoted religious content, such as “@*JesusNarrowWay*”, (see Example 2).

**Table 5:** Sample of tweets and the context of social bot interactions in relation to stance and topic

#	T	Stance	Example tweet
1	A	Against	RT @FollowDMs: follow everyone who retweets this
2	A	Against	RT @JesusNarrowWay: 1 Peter 4:18, If it is hard for the righteous to be saved, what will become of the ungodly and the sinner?
3	HC	Against	RT @VoteHillary2016: donald, are you talking about the 70K votes we lost in 3 states or the nearly 3 million popular votes you lost despite
4	FM	Against	’So @ForgetFeminism according to this...99.99% of the feminists I talk to are NOT “feminists”.ll let them know.#WomenAgainstFeminism’
5	A	Favor	RT @BibleWisdoms: There’s one Lord, one faith, one baptism, and one God and Father of all - Ephesians 4:5-6
6	HC	Favor	RightOn! @Timoniumbill: @ReadyForHillary Mrs. Clean. http://t.co/xBh7FrjZXh #OhHillNo #WakeUpAmerica #StopHillary2016
7	I	Against	RT @cookequipman1: AMERICA’S VET TRAIN #ConnectingAmericanVets #MAGAveteran

**Table 6:** Top bot accounts in indirect interactions for each stance towards the seven topics

T	Favor	Against
A	HaginQuotes, RCSproul, warpawsiraq	2ayaat , lilxstyles, RTAL_D3OAH
CC	Smartassy4ever, jtd_gameon12, bigboater88	AIIAmericanGirI, SassyCon, Moonbattery1
HC	WhatHillaryAte, bluenationuntd, stylebysassys	saynotogop, humoryoulike, UniteBlueSC
FM	geekfeminism, onlyminionquote, tomily4	stopbrutality, FeministShit, SC2TopReplays
LA	succesfultips1, JohnGaltTCMC, SMNW_YRC	TheKeyisPrayer, prolife321, myjesus123
B	UKPollingLive, watching_eu, Brexit_WestMids	moggality, mosthauntedlive, britainsmilhist
I	RealBarcaBooks, RomanCatholic36, umustknowthis1	milagrovargas14, fridayfeeiing,mrmarkel

**The indirect exposure to bots.** We also analyzed bots accounts that were the most predictive of the stance in the EXP features, which were the bots that the users in our datasets were following. Table6 presents the top three bots that influenced the against and in-favor stances for each topic. In general, social media users tended to follow bots that aligned with their leanings. For example, people with an against stance toward atheism tended to follow automated accounts with religious content, such as “@2*ayaat*” and “$@RTAL\_D3OAH.$” The same observation was made for users who supported Hillary, who tended to follow accounts that confirmed their leanings, such as “@*WhatHillaryAte*”; this account is an automated account with retweets and tweets that amplifies content that support to Hillary. Additionally, users with stances that supported the feminist movement followed accounts that promoted the feminist movement, such as “@*geekfeminism*”. As it relates to Brexit supporters, accounts such as “$@Brexit\_WestMids$”, which promoted Brexit through tweets and retweets, was one of the most effective accounts in the friends list to predict the in-favor stance towards Brexit. It is worth noting that, in general, the most effective bots for stance prediction had no direct relation to the topic related to the stance. This can be seen in the top three bots that interacted with users who held against stance toward climate change; these accounts tended to cover a variety of political subjects that had no relation to climate change. For example, the account “@*AIIAmericanGirI*” posted news tweets that were related to the Conservative party . Furthermore, users with an against stance toward Brexit tended to follow bots that distributed content about political news, but had no direct relation to the withdrawal of the United Kingdom from the European Union.

## Inspecting the deleted accounts

On average, the deleted accounts constituted approximately 19% of the overall influential accounts in direct interactions and about 11% of the indirect interactions. One of the limitations in our previous analysis was failing to analyze these deleted accounts. For some of the topics, the number of the deleted accounts in the top 1000 was over 30%; we cannot confirm whether these accounts were bots or real users. This limitation is usually found in the studies of social bot behaviors as a result of collecting tweets in the aftermath of an event (Rizoiu et al. 2018; Luceri et al. 2019; Rizoiu et al. 2018; Shao et al. 2018; Howard and Kollanyi 2016). These deleted accounts have presented a hurdle in many bot-detection studies (Rizoiu et al. 2018; Luceri et al. 2019); as these accounts no longer exist on the Twittersphere, so it was difficult to retrieve the needed information for these accounts to examine the bot behavior of the account. For example, in a study conducted by Luceri et al. (2019), the dataset was composed of approximately 99% suspended accounts. In our work, since we focused on user stances and using the set of influential accounts, the percentage became much lower, compared to previous studies of bots on social networks. The deleted accounts in friends networks (EXP) constituted a much lower percentage in comparison with direct interactions (IN). This is due to the fact that the accounts collected from the direct interactions were extracted from each user’s timeline, which may have contained obsolete mentions, while the friends set tended to only contain the accounts that existed at the time of the collection. In an attempt to overcome part of this limitation, at least as it related to the deleted accounts in the IN features, which had the highest deletion percentage, we decided to manually inspect all the tweets where they were mentioned in the collected users’ timelines; then, based on this tweets, we decided whether they were bots or not. Since this process was time consuming, we considered all the deleted accounts in the top 100 of the influential features of the IN features. As Fig. 3 shows, these trends can be spotted within the first 100 features of the direct interactions.

We used the same annotation guideline of the Varol-2017 Botometer dataset (Varol et al. 2017) to label the deleted accounts as bots or not. The annotation guideline of the bot-detection study by Varol et al. (2017) was based on inspecting the account’s profile page and looking for common flags, such as a stock profile image or retweeting that occurs within seconds. As these deleted accounts had no Twitter profile information, inspecting these accounts’ profile pages was not applicable in our case. Since there was no unified rule to label an account as a bot, we further retrieved a set of tweets from stance dataset where the users interacted with these deleted accounts. These tweets provided additional clues to label the set of deleted accounts and inspect their behavior.

After manual inspection, we found that some of those accounts were likely bots. Table 7 presents the amount of existing bots and deleted bots in the top 100 IN features. In general, bots constituted less than 11% of the top 100 features of in-favor and against stances. The topic of immigration had the most proportion of bots that interacted with the in-favor stance. As it related to against stances, the legalization of abortion and atheism contained the largest amount of bots, compared to other topics.

Table 8 shows a sample of tweets that demonstrated the characteristics of the the deleted accounts. Some of the deleted accounts had the term “bot” as part of the user name, such as in Example 3. In other cases, the account name indicates the behavior of the account, such as “$@theism\_sucks$” (see Examples 4 and 5). User interactions with this account were conducted in the sense of mentioning it to defend their religious perspectives. Some accounts had limited content in our dataset such as in Example 6. In this example, the account “@*BarbietheBrain*” appeared in a retweet with other accounts as a means of promoting these accounts. We considered such situations as promoting an account by spreading automated content and labeled the account as a bot.^17^ Other deleted accounts had tweets that showed somewhat personal messages such as in Example 7. The account “@*coolredmac*” was a suspended account, as this account had hateful tweets, such as those in Example 8. In this case, we labeled this account as not being a bot account. Other accounts had normal content based on the retweet behavior in our dataset, such as in Example 9. The account “@*PETTYMAMII*” was a suspended account which has non-hateful tweets when we retrieved the account’s timeline tweets from our dataset.

One of the obvious indicators of bot behavior was the vast amount of retweets that showed the content of the account, such as those in Examples 10, 11, and 12. These examples showed retweets from a bot account “@*LiveActionFilms*” interacting with against stance on the legalization of abortion. This account was suspended because of spreading negative messages.

**Table 7:** The number of deleted accounts and the expected bots in the top 100 influential accounts on stance prediction

T	Favor				Against			
	Deleted	Deleted bots	Existing bots	Total bots	Deleted	Deleted bots	Existing bots	total
A	8	2	3	5	20	8	0	8
CC	8	2	2	4	15	0	0	0
HC	33	8	1	9	20	2	2	4
FM	23	1	1	2	27	2	5	7
LA	25	4	5	9	10	3	5	8
B	12	5	2	7	9	2	5	7
I	8	3	7	10	7	3	4	7

**Table 8:** Sample of tweets that interacted with deleted accounts in the top 100 features of (IN)

	T	S	Type	Tweet
1	CC	–	NotBot	@_PinealGland: 1984 https://pic.twitter.com/DgmniSvYON” one of the best books ever
2	HC	–	Bot	@srtalbot2 http://t.co/fjr9IeSKak
3	A	–	Bot	RT @ArchbishopYoung: “Today is your day, your mountain is waiting, so get on your way.” - Dr Seuss #Quote
4	A	+	Bot	@X @theism_sucks we christians dont want dark matters to rule our world. we love the #Light #happiness #God’
5	A	+	Bot	@2ManyOfUs @theism_sucks pettiness? #bible speaks the truth. #owned again #atheist sucks LOL’
6	B	+	Bot	RT @*Silentwoo*: @*IrishVol*69*th* @*alley*167 @*BarbietheBrain* @*LisaNiebs* @*NeensCa* @*heyitsCarolyn* @*Sekusa*1 @*ON*11.
7	B	+	NotBot	@*X* @*coolredmac* Well said, Sir.
8	B	+	NotBot	RT @*coolredmac*: Which is why she is no longer prime minister Emmanuel Macron praises Theresa May for being loyal and respectful to EU
9	FM	+	NotBot	RT @*PETTYMAMII*: Not seeing your bestfriend for a long X time really hurts
10	LA	–	Bot	RT @*LiveActionFilms*: Paul: “The right to life and freedom of religion preexist government.” #VVS14 #prolife’
11	LA	–	Bot	RT @*LiveActionFilms*: “Humanizing,” @PBS? What is human, anyway? Watch the video:X #AfterTiller #abortionaccess’
12	LA	–	Bot	RT @*LiveActionFilms*: Our latest video showing @*PPact* dangerous #SexEd for kids was featured on OReilly last night”

We used “X” to mask some users accounts and hide sensitive content

## Verifying the bot/non bot accounts

In order to verify the reliability of Botometer in detecting bots and non-bot accounts, we verified the propriety of the top 10 accounts for each topic/stance and identified the likely bot accounts. We inspect the type of the accounts and measure the Cohen’s kappa score between the Botometer and annotation labels to gauge the reliability between the two labels. We used the same annotation guideline of the Varol-2017  (Varol et al. 2017) to verify the likelihood of bot. Also, we used Bot-Detective API (Kouvela et al. 2020) to provide further explainable hints for bot-like accounts, that helps us to provide ground truth labels by using extra information beyond inspecting Twitter page. Table 9 provides some examples of accounts and examples of explanations provided by Bot-Detective in identifying the bot-likely accounts. We found a high alignment between manual annotation and Botometer labels in identifying human accounts with Cohen’s kappa score equal to 68.81%, which indicates a substantial agreement between the manual annotations and Botometer. Even in cases where the account seems to be a nonpersonal account, using Bot-Detective helps in verifying those accounts. For instance, the account @*hqtriviafans* is a fan page for the trivia game show. Example 3 in Table 9, shows that this account has a high likelihood to be human as the average number of characters per tweet (72.75 ). Bots usually have 143.7 characters on their tweets. Although, for some bot accounts, the score was on edge, even for the annotators. This is due to the fact that some of these accounts are mostly having low tweets with the default setting. In these cases, we use Bot-Detective to provide explanations based on non-profile information and further inspect the type of those accounts. For instance, the account @*GOTGeekX*, has a score of 3.7 out of 5 using Bot-Detective, and 0.96 in Botometer. Using the explanations generated by Bot-Detective, the account is highly likely a bot, considering that the URL per word ratio for each tweet is suspiciously high (Table 9, example 2).

**Table 9:** Sample of accounts that we verified their bot likeness with explanation from Bot-Detective tool

	Type	V	Account with explanation
1	NotBot	$\checkmark$	@RichardDawkins This account is verified. Almost always, this means that the account belongs to a non-bot user.
2	Bot	$\checkmark$	@GOTGeekX This account’s URL per word ratio for each tweet, is suspiciously high
3	NotBot	$\checkmark$	@hqtriviafans Normal average number of characters per tweet (72.75 ). Bots usually have 143.7 characters on their tweets
4	Bot	✗	@laura_beene the average liked tweets is normal
5	Bot	✗	@saysmysister This account uses symbols rarely (11.53 symbols per tweet). Bots usually have 21.2 symbols per tweet, on average

(V) indicates the verification of likeness

## Discussion

Given the prevalence of bots in social media, it is crucial to examine the role these accounts play in affecting the online users’ stances and to understand the interaction behavior of these accounts. Measuring the factors that relate to people’s stances in social media is a complex process that is influenced by various behavioral signals (Lee et al. 2010; Cha et al. 2010) Motivated by this challenge, we investigated the role of bots using a gold-standard stance-labeled dataset that contained real users’ stances on seven topics; this dataset contained events and topics that covered three main domains (i.e., politics, religion, and social aspects). In this study, we extended our understanding of the relationship between bots and the stances of social media users, and we highlighted various implications for bot studies.

### Bot and human presence on stances

To answer the first research question and to assess the association between bots and stance, we analyzed the most influential accounts to predict users’ stances, and we inspected the presence of bots among these accounts. We showed bot and human distributions within the top 1000 most predictive accounts for their stances concerning seven topics.

Overall, while bot accounts were present in the top influential accounts in predicting the stances thereof, the bots had the lowest percentage, compared to human accounts, as shown in Fig. 2. This result while it confirms our first hypothesis (H1) where bots have a presence in the top features, this presence is considered much less than what we expected. This finding places an emphasis on the noticeable connection between human accounts and a given stance, compared to that of bots. Our results align with the recent study by Dunn et al. (2020) who investigate the effect of bots in comparison with people in social media dataset related to COVID19. They found the role of bots in spreading fake news about antivaccine is limited.

Moreover, we showed the magnitude of the effect of the top 1000 accounts on predicting the stances related to three kinds of real user accounts (i.e., normal, famous, and ultra-famous), as shown in Fig. 3. The noticeable link between the *ultra-famous* accounts and stance formation can be observed in the first top 10 accounts that influenced the given stance. This finding does not align with the “million followers fallacy” theory (Avnit 2009), which was confirmed for Twitter by Cha et al. (2010). Throughout this study, we showed that the generalization of the followers’ theory is not applicable in the realm of measuring the influence and connection to stance.

**Fig. 7:**
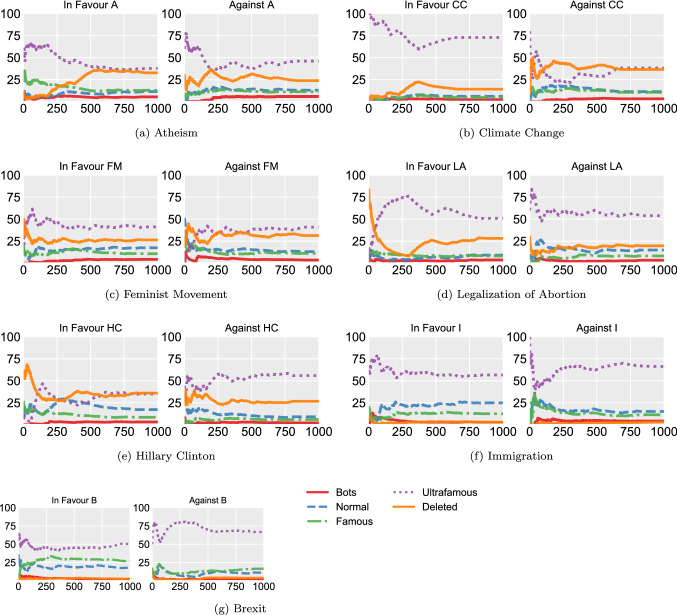
The distribution of bots on the top 1000 influential accounts from the direct interactions (IN) in predicting the against/favor stance (topic level)

**Fig. 8:**
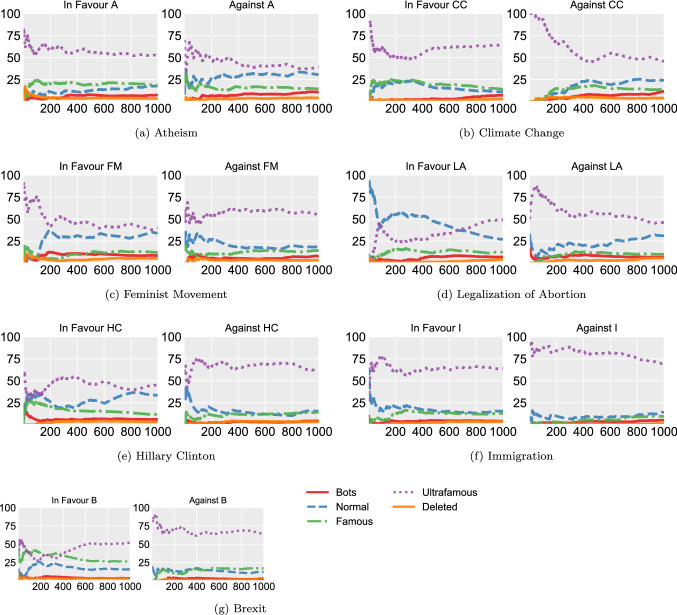
The distribution of bots on the top 1000 influential accounts from indirect exposure (EXP) in predicting the against/favor stance (topic level)

Furthermore, we provide a finer granularity analysis of the role of the bots on the topic level (see Figs. 8 and 7 in Appendix A). It can be observed that the relationship between the ultra-famous users and the given stance represented the general trend on the topic level.

### The link between bots and supporting versus opposing stances

When addressing the second research question, we noticed that the role of bots on the supporting and opposing stances was relatively different for a majority of the topics, which aligns to our second hypothesis (H2). This can be seen in the proportion of bots that influenced the stances, as shown in Fig. 2, even though the bots presence in the two topics of atheism and climate change was sizable on the against stance, compared to the in-favor stance. However, by inspecting the bots in the friends set (EXP) for climate change, we found that most of these automated accounts had no direct relation to climate change. As for the other topics, there was at least one bot account in the top three accounts in the friends set that were related to the topic of the stance. This finding indicates that bots can have a greater role on a specific stance type than the others for some topics . For instance, users with stances that oppose atheism tend to follow and interact with bots that have religious content.

The same was observed for the friends accounts (EXP) that influenced the against and in-support stances towards the legalization of abortion, which had approximately 7% bots. Furthermore, there was a noticeable difference in bot distribution at the topic level. The presence of bot accounts was sizable in the direct interaction (IN) and indirect exposure (EXP) , in atheism, as is shown in Fig. 2. The fewest bot accounts were seen in Brexit, where bots constituted approximately 2% of the overall interactions. When we further inspected the type of bots that influenced the stance toward atheism, we noticed that these accounts had a religious theme that promoted faith, which supported the specific type of stance. Similarly, the bots that influenced Brexit stances tended to have a political theme and a focus on news related to the withdrawal of the United Kingdom from the European Union, such as $watching\_eu$ and $Brexit\_WestMids$.

Moreover, we inspected the stance level of interactions with bots. We showed that users tended to directly interact with bots that had a stance that was different from theirs (see Examples 3 and 5 and Table 5). This indicates the simple direct interaction to a bot’s content does not have a direct relation to a user’s stance. This behavior can be supported by the backfire effect (Nyhan and Reifler 2010), which means that usually exposure to this kind of contradicting content leads to maintaining a stance despite new information that firmly contradicts it. This finding helps with gaining a better understanding of the effect of social bots on users’ stances on social media. Also, it is worth noting that in general social media users tend to follow bots that align with their leanings. This can be seen in the top accounts that influenced the prediction of against stance towards atheism, where users tended to follow religious accounts, such as 2*ayaat* and $RTAL\_D3OAH$, as shown in Table 6. Nevertheless, the general trend was that the top bots accounts in the friends list had no direct relation to the stance topic. This can be seen in the most influential follower bot accounts in predicting an against stance toward climate change. One of the top bot accounts was *AIIAmericanGirI*, which had no direct relation to climate change, as is shown in Table 6.

### Bots’ link to stance based on the interactions type

The third research question was concerned with whether users were influenced by being exposed to posts from the social bots. We extended the effort of previous research in this field by looking beyond the bots diffusion and analyzing bot interplay with online stances using two kinds of networks: direct interactions with bots (IN) and indirect exposure to their content (EXP). Overall, we found that users’ stances were more related to bots whose content they were exposed to by following them than by directly interacting with them through retweets, replies, or mentions. This finding supports the third hypothesis (H3) where bots are shown to have presence in the direct and indirect interactions, which shows that they can affect stances even indirectly by having the user get exposed to their posted content without the need to interact directly with them.

Furthermore, we found that users with an against stance towards a given topic tended to have more indirect interactions with bots, compared to direct interactions related to the same stance toward a topic. This kind of online behavior places an emphasis on the potential hidden effect of bots, which contrasts with the existing norms of studying the effect of social bots by solely focusing on the direct interactions of users with the bot content “retweets”.

### Implications

Prior to this study, the literature has informed us that bots are present in social media, and they affect drifting discussions and spreading certain information related to a given topic. However, it was not clear if their presence have any relation with users’ stance online. Our main findings suggested that bots’ presence is linked to stance as it can be correlated to the main signals that can predict a given stance. However, our analysis shows that bots role is minimal compared to influential and famous human accounts. This finding of our study suggests that the large fear of bots spreading harmful messages on social media might be overrated. This study does not deny the effect of their presence on the stances of people on a given topic, but we show that it is marginal compared to other factors. Our findings in this study set the path for the research community with future research opportunities to further examine the clear impact of bots on people stances by conducting qualitative studies.

Another implication should be geared towards implementing the policy of social media platforms, such as Twitter, when dealing with these accounts. It is important to increase the awareness of social media users about the effect of bots. As it has been shown, having users exposed to bots content through following them is enough to predict their stance, even more than when users interact directly with bots content through retweeting or commenting.

Finally, stance detection on social media can enable a thorough understanding of the interplay between stance towards a topic and the online signals. The ability to further analyze the hidden effect of bots as indirect interactions presents new territory for the current study of automated accounts amplification of fake news towards a certain stance (supporting/against). The focus of these studies needs to further address the indirect interactions instead of solo dependence on direct interactions as a retweet.

### Limitations

Understanding bots’ effect on social media is one of the highly valued questions in social computing community (Abokhodair et al. 2015; Zheng et al. 2019; Seering et al. 2018). However, it is challenging to study this kind of effect on the online users’ stance. In our study, we used stance detection as the mean to link bots effect on users’ stance by inspecting if those bots can act as predictive features to the stance. However, one limitation of our approach is that it is hard to confirm that detecting predictive bots for users’ stance means that the stance has been affected by the bot not the other way around, that they interact/follow those bots since they have this stance and those bots reinforce their leaning. This is the very typical “correlation does not mean causality” problem (DeMarie-Dreblow 1991). This is a common limitation even in existing studies that identify the bots’ effect by analyzing their spread within OSNs (Aiello et al. 2012; Schuchard et al. 2019). Nevertheless, either their effect is by shaping users’ stance or by reinforcing an existing stance, both still show that bots do have some role in link with polarised stances to the level that they can become predictive signals for a given stance.

Another well-known limitation on studying bots behaviour in the social network is the deleted accounts in the collected dataset. In our work, we tried to address this limitation by inspecting some of those accounts manually. However, our addressing to the problem has its other limitations by itself, since we decided an account to be bot or not based on limited signals from the tweets mentioning them in the users’ timelines rather than having a proper analysis to their profiles (that do not exist anymore). Unfortunately, this will remain an issue that is difficult to resolve. Nevertheless, we hope that our manual inspection to the deleted accounts gives some indication about these accounts overall behaviour.

## Conclusion

In this study, we sought to understand the contemporary debate—admittedly, bots are everywhere, but what is the role that bots play related to polarized stances? We investigated this question by examining two kinds of online user interactions: direct interactions and indirect exposure. For the direct interactions, we evaluated users’ interactions with bots with the use of mentions. As it related to indirect exposure, the analysis was carried out on the friends set of users to examine their exposure to bot content. We used the gold standard of annotated stance data that contains seven topics covering politics, religion, and social aspects. We showed empirical evidence of the effect of social bots on specific stances by using the state-of-the-art stance-detection model.

Our findings further indicate that users on social media tended to have limited direct interactions with social bots, that famous users in terms of followers had a sizable relationship with these stances, and that ultra-famous users tended to have the most presence on the stances interactions of specific topics from various domains. Moreover, social media users had indirect exposure to bots, compared to direct interaction, which suggests that users are more exposed to bot content in an indirect manner by following these accounts, compared to direct interaction by retweets or mentions. These findings help to extend the understanding of the effect of bots on stances on social networks. In the future, further analysis of the bots’ temporal interactions with specific stances would help to further understand the overall effect of bot accounts on online stances.

## Figures and Tables

**Fig. 5:**
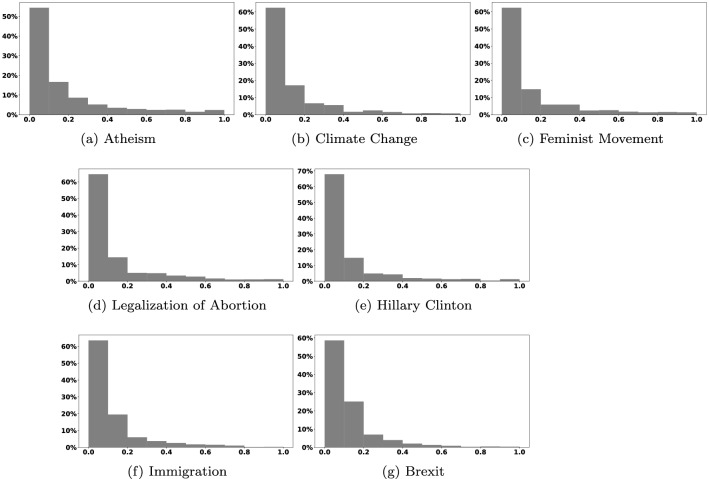
The distribution of accounts scores on the top 1000 influential accounts from direct interactions (IN) in predicting the against/favor stance (Topic level)

**Fig. 6:**
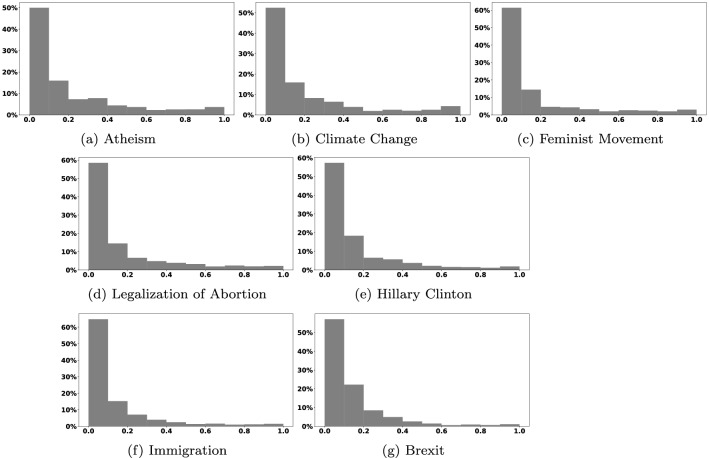
The distribution of accounts scores on the top 1000 influential accounts from in direct interactions (EXP) in predicting the against/favor stance (topic level)

## F1-score equations

The macro $\hbox {F}_{{avg}}$ was calculated as presented in Equation 1:

2 $$\begin{aligned} F_{avg} = \frac{F_{favor} + F_{against}}{2} \end{aligned}$$

where $\hbox {F}_{{favor}}$ and $\hbox {F}_{{against}}$ are calculated as shown below:

3 $$\begin{aligned} F_{favor}= & {} \frac{2P_{favor} R_{favor}}{P_{favor}+R_{favor}} \end{aligned}$$

4 $$\begin{aligned} F_{against}= & {} \frac{2P_{against} R_{against}}{P_{against}+R_{against}} \end{aligned}$$

## Distribution of the accounts scores on topic level

See Figs. 5 and 6.

## Chi-squared test for accounts distributions

The chi-squared test results for each topic (Table 10).

**Table 10:** Chi-squared test for accounts distributions between IN and EXP bot accounts

**T**	**Favor**	**Against**
A	1.69e–27***	1.7e–33***
CC	1.96e–06***	1.11e–65***
HC	5.43e–34***	5.28e–44***
FM	7.60e–12***	3.03e–54***
LA	3.02e–17***	2.45e–17***
B	0.56	0.56
I	0.36	0.046*
Overall	3.17e–69***	1.77e–178***

**p*<0.05; ***p*<0.01; ****p*<0.001

## Distribution of baseline tweets in the dataset

See Table 11

**Table 11:** The distribution of baseline tweets per topic in the SemEval and Events datasets

Dataset	Topic	Favor	Against	Neither	Total
SemEval	Atheism (A)	93	349	108	550
	Climate change (CC)	284	16	161	461
	Hillary clinton (HC)	113	387	170	670
	Feminist movement (FM)	171	245	108	524
	Legalization of abortion (LA)	98	406	166	670
Events	Brexit (B)	71	395	0	466
	Immigrations (I)	232	1280	0	1512
	Total	1062	3078	713	4853

## Footnotes

1 “bots” will be used henceforth to refer to “social bots”.

2 We refer to Twitter as “social media” in this paper.

3 Polarised stance refers to non-neutral stances. It does not necessarily imply extreme stance, but just being either against or in-favor of a given topic.

4 More examples of interesting and creative online bots are available on Botwiki: https://botwiki.org/.

5 https://help.twitter.com/en/rules-and-policies/twitter-automation.

6 Previously know as Figure Eight and CrowdFlower. https://appen.com/.

7 https://success.appen.com/hc/en-us/articles/208465816-Quality-Management.

8 https://developer.twitter.com/en/docs/tweets/timelines/api-reference/get-statuses-home_timeline.

9 https://developer.twitter.com/en/docs/accounts-and-users/follow-search-get-users/api-reference/get-friends-list.

10 Since our focus in this study is to provide a through analysis of the interplay between stance and bot advised by the most well-known benchmark stance dataset we kept the Climate change topic in our analysis even when it has the lowest F1 score.

11 https://botometer.iuni.iu.edu/.

12 https://help.twitter.com/en/managing-your-account/suspended-twitter-accounts.

13 https://sysomos.com/inside-twitter/twitter-statistics/.

14 Appendix B shows the Botometer scores distribution on topic level

15 We also examined a threshold of 0.5, but found that it increased misclassification of the human accounts as bots without improving the detection of new bots.

16 https://cnets.indiana.edu/blog/2020/09/01/botometer-v4/.

17 In Example 6, a combined account with “@*BarbietheBrain*” posed bot behavior based on their profile characteristics.

## References

[CR1] Abokhodair N, Yoo D, McDonald DW (2015) Dissecting a social botnet: growth, content and influence in twitter. In: Proceedings of the 18th ACM conference on computer supported cooperative work social computing, CSCW’15, New York, NY, USA. Association for Computing Machinery, pp 839-851. ISBN 9781450329224. 10.1145/2675133.2675208

[CR2] Abu-El-Rub N, Mueen A (2019) Botcamp: Bot-driven interactions in social campaigns. In: The world wide web conference. ACM, pp 2529–2535

[CR3] Aiello LM, Deplano M, Schifanella R, Ruffo G (2012) People are strange when you’re a stranger: impact and influence of bots on social networks. In: Sixth international AAAI conference on weblogs and social media

[CR4] AlDayel A, Magdy W (2019a) Assessing sentiment of the expressed stance on social media. In: Proceedings of the 11th international conference on social informatics (SocInfo 2019)

[CR5] AlDayel A, Magdy W (2019b) Your stance is exposed! analysing possible factors for stance detection on social media. In: The 22nd ACM conference on computer-supported cooperative work and social computing. ACM

[CR6] AlDayel A, Magdy W (2021). Stance detection on social media: state of the art and trends. Inf Process Manag.

[CR7] Allcott H, Gentzkow M (2017) Social media and fake news in the 2016 election. Technical report, National Bureau of Economic Research

[CR8] Avnit A (2009) The million followers fallacy. Pravda Media Group

[CR9] Bastos MT, Mercea D (2019). The brexit botnet and user-generated hyperpartisan news. Soc Sci Comput Rev.

[CR10] Bessi A, Ferrara E (2016) Social bots distort the 2016 us presidential election online discussion. First Monday, 21 (11–7)

[CR11] Boichak O, Jackson S, Hemsley J, Tanupabrungsun S (2018) Automated diffusion? bots and their influence during the 2016 us presidential election. In: International conference on information. Springer, pp 17–26

[CR12] Broniatowski DA, Jamison AM, Qi S, AlKulaib L, Chen T, Benton A, Quinn SC, Dredze M (2018). Weaponized health communication: Twitter bots and russian trolls amplify the vaccine debate. Am J Public Health.

[CR13] Center PR (2013) News use across social media platforms

[CR14] Cha M, Haddadi H, Benevenuto F, Gummadi KP (2010) Measuring user influence in twitter: the million follower fallacy. In: Fourth international AAAI conference on weblogs and social media

[CR15] Cossu J-V, Labatut V, Dugué N (2016). A review of features for the discrimination of twitter users: application to the prediction of offline influence. Soc Netw Anal Min.

[CR16] Cresci S, Di Pietro R, Petrocchi M, Spognardi A, Tesconi M (2015). Fame for sale: efficient detection of fake twitter followers. Decis Support Syst.

[CR17] Darwish K, Magdy W, Zanouda T (2017) Trump vs. hillary: What went viral during the 2016 us presidential election. In: International conference on social informatics. Springer, pp 143–161

[CR18] Darwish K, Stefanov P, Aupetit MJ, Nakov P (2019) Unsupervised user stance detection on twitter. In: ICWSM

[CR19] Davis CA, Varol O, Ferrara E, Flammini A, Menczer F (2016) Botornot: A system to evaluate social bots. In: Proceedings of the 25th international conference companion on world wide web. International world wide web conferences steering committee, 2016, pp 273–274

[CR20] DeMarie-Dreblow D (1991). Relation between knowledge and memory: a reminder that correlation does not imply causality. Child Dev.

[CR21] Dunn AG, Surian D, Dalmazzo J, Rezazadegan D, Steffens M, Dyda A, Leask J, Coiera E, Dey A, Mandl KD (2020). Limited role of bots in spreading vaccine-critical information among active twitter users in the united states: 2017–2019. Am J Public Health.

[CR22] Dutta HS, Aggarwal K, Chakraborty T (2021) Decife: detecting collusive users involved in blackmarket following services on twitter. In: Proceedings of the 32nd ACM conference on hypertext and social media, pp 91–100

[CR23] Ferrara E (2017) Disinformation and social bot operations in the run up to the 2017 French presidential election. First Monday 22(8)

[CR24] Ferrara E (2019) Bots, elections, and social media: a brief overview. arXiv:1910.01720

[CR25] Friedkin NE, Johnsen EC (1990). Social influence and opinions. J Math Sociol.

[CR26] Garimella K, West R (2019) Hot streaks on social media. In: Proceedings of the international AAAI conference on web and social media, vol 13, No. 01. pp 170–180. https://www.aaai.org/ojs/index.php/ICWSM/article/view/3219

[CR27] Gilani Z, Farahbakhsh R, Tyson G, Crowcroft J (2019). A large-scale behavioural analysis of bots and humans on twitter. ACM Trans Web.

[CR28] Graells-Garrido E, Baeza-Yates R, Lalmas M (2020) Every colour you are: stance prediction and turnaround in controversial issues. WebSci

[CR29] Grčar M, Cherepnalkoski D, Mozetič I, Novak PK (2017). Stance and influence of twitter users regarding the brexit referendum. Comput Soc Netw.

[CR30] Hegelich S, Janetzko D (2016) Are social bots on twitter political actors? empirical evidence from a Ukrainian social botnet. In: Tenth international AAAI conference on web and social media

[CR31] Howard PN, Kollanyi B (2016) Bots,# strongerin, and# brexit: computational propaganda during the uk-eu referendum. Available at SSRN 2798311

[CR32] Kouvela M, Dimitriadis I, Vakali A, Bot-detective: an explainable twitter bot detection service with crowdsourcing functionalities. In: Proceedings of the 12th international conference on management of digital ecosystems, MEDES’20, New York, NY, USA. Association for Computing Machinery, pp 55–63. ISBN 9781450381154. 10.1145/3415958.3433075

[CR33] Lai M, Patti V, Ruffo G, Rosso P (2018) Stance evolution and twitter interactions in an Italian political debate. In: International conference on applications of natural language to information systems. Springer, pp 15–27

[CR34] Lee C, Kwak H, Park H, Moon S (2010) Finding influentials based on the temporal order of information adoption in twitter. In: Proceedings of the 19th international conference on World wide web. ACM, pp 1137–1138

[CR35] Luceri L, Deb A, Badawy A, Ferrara E (2019) Red bots do it better: comparative analysis of social bot partisan behavior. In: Companion proceedings of the 2019 world wide web conference. ACM, pp 1007–1012

[CR36] Magdy W, Darwish K, Abokhodair N, Rahimi A, Baldwin T (2016a) # isisisnotislam or# deportallmuslims?: predicting unspoken views. In: Proceedings of the 8th ACM conference on web science. ACM, pp 95–106

[CR37] Magdy W, Darwish K, Abokhodair N, Rahimi A, Baldwin T (2016b) # isisisnotislam or# deportallmuslims?: predicting unspoken views. In: Proceedings of the 8th ACM conference on web science. ACM, , pp 95–106. ISBN 1-4503-4208-6

[CR38] Mendoza M, Tesconi M, Cresci S (2020). Bots in social and interaction networks: detection and impact estimation. ACM Trans Inf Syst (TOIS).

[CR39] Mohammad S, Kiritchenko S, Sobhani P, Zhu X-D, Cherry C (2016) SemEval-2016 Task 6: detecting Stance in Tweets. In: SemEval@ NAACL-HLT, pp 31–41

[CR40] Musco C, Musco C, Tsourakakis CE (2018) Minimizing polarization and disagreement in social networks. In: Proceedings of the 2018 world wide web conference, pp 369–378

[CR41] Ng LHX, Carley K (2021) Flipping stance: social influence on bot’s and non bot’s Covid vaccine stance. MIS2 workshop at KDD 2021

[CR42] Nyhan B, Reifler J (2010). When corrections fail: the persistence of political misperceptions. Polit Behav.

[CR43] Puertas E, Moreno-Sandoval LG, Plaza-del Arco FM, Alvarado-Valencia JA, Pomares-Quimbaya A, Alfonso L (2019) Bots and gender profiling on twitter using sociolinguistic features

[CR44] Pulido CM, Redondo-Sama G, Sordé-Martí T, Flecha R (2018). Social impact in social media: a new method to evaluate the social impact of research. PLoS ONE.

[CR45] Ratkiewicz J, Conover M, Meiss M, Gonçalves B, Patil S, Flammini A, Menczer F, Detecting and tracking the spread of astroturf memes in microblog streams

[CR46] Ratkiewicz J, Conover M, Meiss M, Gonçalves B, Patil S, Flammini A, Menczer F (2011) Truthy: mapping the spread of astroturf in microblog streams. In: Proceedings of the 20th international conference companion on world wide web, WWW ’11, New York, NY, USA. Association for Computing Machinery, pp 249–252. ISBN 9781450306379. 10.1145/1963192.1963301

[CR47] Rizoiu M-A, Graham T, Zhang R, Zhang Y, Ackland R, Xie L (2018) # debatenight: the role and influence of socialbots on twitter during the 1st 2016 us presidential debate. In: Twelfth international AAAI conference on web and social media

[CR48] Samih Y, Darwish K (2020) A few topical tweets are enough for effective user-level stance detection. arXiv:2004.03485

[CR49] Santia GC, Mujib MI, Williams JR (2019) Detecting social bots on facebook in an information veracity context. In: Proceedings of the international AAAI conference on web and social media, vol 13, pp 463–472

[CR50] Schuchard R, Crooks AT, Stefanidis A, Croitoru A (2019). Bot stamina: examining the influence and staying power of bots in online social networks. Appl Netw Sci.

[CR51] Seering J, Flores JP, Savage S, Hammer J (2018). The social roles of bots: evaluating impact of bots on discussions in online communities. Proc ACM Hum Comput Interact.

[CR52] Shao C, Ciampaglia GL, Varol O, Yang K-C, Flammini A, Menczer F (2018). The spread of low-credibility content by social bots. Nat Commun.

[CR53] Stella M, Ferrara E, De Domenico M (2018). Bots increase exposure to negative and inflammatory content in online social systems. Proc Natl Acad Sci.

[CR54] Tardelli S, Avvenuti M, Tesconi M, Cresci S Characterizing social bots spreading financial disinformation. In: Meiselwitz G (ed) Social computing and social media. Design, ethics, user behavior, and social network analysis. Springer, pp 376–392. ISBN 978-3-030-49570-1

[CR55] Thonet T, Cabanac G, Boughanem M, Pinel-Sauvagnat K (2017) Users are known by the company they keep: topic models for viewpoint discovery in social networks. In: Proceedings of the 2017 ACM on conference on information and knowledge management. ACM, pp 87–96. 10.1145/3132847.3132897.

[CR56] Varol O, Ferrara E, Davis CA, Menczer F, Flammini A (2017) Online human-bot interactions: detection, estimation, and characterization. In: Eleventh international AAAI conference on web and social media

[CR57] Wate Y (2021) 10 best twitter bots you should follow in 2022. TechPP URL https://techpp.com/2021/12/10/best-twitter-bots/

[CR58] Yang K-C, Varol O, Davis CA, Ferrara E, Flammini A, Menczer F (2019). Arming the public with artificial intelligence to counter social bots. Human Behav Emerg Technol.

[CR59] Zheng LN, Albano CM, Vora NM, Mai F, Nickerson JV (2019). The roles bots play in Wikipedia. Proc ACM Hum Comput Interact.

